# A Cognitive Behavioral Therapy–Based Text Messaging Intervention Versus Medical Management for HIV-Infected Substance Users: Study Protocol for a Pilot Randomized Trial

**DOI:** 10.2196/resprot.5407

**Published:** 2016-06-24

**Authors:** Suzette Glasner-Edwards, Kevin Patrick, Michele L Ybarra, Cathy J Reback, Richard A Rawson, Helene Chokron Garneau, Kathryn Chavez, Alexandra Venegas

**Affiliations:** ^1^ Integrated Substance Abuse Programs Semel Institute for Neuroscience and Human Behavior University of California Los Angeles Los Angeles, CA United States; ^2^ University of California at San Diego La Jolla, CA United States; ^3^ Center for Innovative Public Health Research San Clemente, CA United States; ^4^ Friends Research Institute Los Angeles, CA United States

**Keywords:** SMS, medication adherence, HIV, relapse prevention, text messaging, CBT, ART

## Abstract

**Background:**

Evidence-based psychosocial interventions for addictions and related conditions such as cognitive behavioral therapy (CBT) are underutilized. Obstacles to implementation of CBT in clinical settings include limited availability of quality training, supervision, and certification in CBT for clinicians; high rates of clinician turnover and high caseloads; and limited qualifications of the workforce to facilitate CBT expertise.

**Objective:**

Mobile phone–based delivery of CBT, if demonstrated to be feasible and effective, could be transformative in broadening its application and improving the quality of addiction treatment. No experimental interventions that deliver CBT targeting both drug use and medication adherence using text messaging have been previously reported; as such, the objective of this study is to develop and test an SMS-based treatment program for HIV-positive adults with comorbid substance use disorders.

**Methods:**

With user input, we developed a 12-week CBT-based text messaging intervention (TXT-CBT) targeting antiretroviral (ART) adherence, risk behaviors, and drug use in a population of HIV-infected substance users.

**Results:**

The intervention has been developed and is presently being tested in a pilot randomized clinical trial. Results will be reported later this year.

**Conclusions:**

This investigation will yield valuable knowledge about the utility of a cost-effective, readily deployable text messaging behavioral intervention for HIV-infected drug users.

## Introduction

### Injection Drug Use and HIV

Injection drug use is a major risk factor for HIV infection, and people who inject drugs (PWID) account for a substantial proportion of new HIV infections in the Unites States and more than one-third of new AIDS cases (a proportion nearly double that of 10 years ago) [1]. This is not surprising given that the risk of infection after injection with an HIV-contaminated syringe is estimated to be 0.4% to 2.4% (median 0.8%; approximately 1 in 125 injections) [2].

Sharing contaminated needles and other injection equipment among PWID is a known source of the increased incidence of HIV transmission in this population, and PWID with sexual risk behaviors are at heightened risk for HIV [3,4]. New and easily deployable interventions targeting the most vulnerable individuals are urgently needed to reduce HIV transmission [5]. The goal of the present research is to develop and evaluate a cost-effective and novel technology-based approach for treating drug dependence and associated HIV risk and treatment adherence problems.

### Reducing HIV Risk Behaviors and Improving HIV Treatment Regimen Adherence

#### ART Adherence Among HIV Positive Drug Users

Among the most promising interventions to address drug dependence, associated HIV risk behaviors, and injection-related HIV transmission are counseling to decrease the number of injections by treating the underlying drug dependence [6] and antiretroviral treatment (ART) to reduce viral load and diminish the likelihood of HIV transmission in the face of exposure for those who are HIV infected [7].

There is ample evidence suggesting that HIV-infected PWID are less likely to access HIV treatment and that once treatment is initiated, they are less likely to be adherent than former and nondrug users [8,9]. ART adherence is critically important; suboptimal dosing can contribute to the development of medication resistance and result in negative consequences including rebounding of HIV RNA levels, sometimes to above baseline levels [10,11]. Less than 5% of PWID receive CD4 cell count monitoring at a frequency consistent with clinical recommendations [12]. Nevertheless, PWID who adhere to antiretroviral therapies have HIV outcomes that are comparable to non-PWID [7]. Preliminary studies suggest that cognitive behavioral therapy (CBT)–based ART adherence counseling (Life-Steps) [11,13,14] is effective among HIV-positive drug users [15].

#### Cognitive Behavioral Therapy for Substance Use Disorders

Both behavioral and cognitive-behavioral treatment approaches have therapeutic effects on a range of functional outcomes among adults with drug use disorders [16]. Although CBT has been evaluated empirically for the treatment of drug users, no studies to date have used mobile phone technology to deliver this intervention to drug-dependent populations.

CBT is among the most widely studied psychosocial interventions for substance users in well-controlled randomized trials. The therapeutic effects of CBT are robust and have been well established across various substance using populations, including those who are dependent on opioids [17-20], marijuana [21], alcohol [22], and stimulants [23]. Based upon social learning theory, a central assumption of CBT treatment is that substance dependence emerges from a process whereby the individual learns through experience about the reinforcement value of the substance [24]. Anticipated reinforcement is thought to drive continued and problematic behavioral patterns of substance use. CBT therefore focuses on the goal of facilitating abstinence from substance use by teaching new alternative, reinforcing behaviors.

In addition to its efficacy in reducing substance use, effects of CBT on HIV risk behaviors have also been reported [6,25]. Pinkerton [25] compared the effectiveness of a 7-session CBT group emphasizing motivation, skills, and self-efficacy related to HIV risk reduction relative to a single video-based HIV/AIDS education session in 3706 high-risk men and women. Both interventions were effective in reducing sexual risk behaviors, but the reduction was much higher with CBT on a number of indices. In particular, CBT participants reduced their mean number of acts of unprotected sexual intercourse from 24.7 at baseline to 12.0 at one-year follow-up, whereas those in the video intervention condition reduced from a mean of 23.9 acts to 16.7 at follow-up. Schroeder [26] reported comparable reductions in sexual and drug-related HIV risk behaviors among cocaine users receiving opioid substitution therapy (OST) concurrently with CBT or contingency management. In both of the psychosocial conditions, participants received education concerning HIV transmission and risk reduction practices. These results suggest broad beneficial effects of OST augmented with behavioral interventions for reducing HIV risk behaviors. As such, the experimental CBT-based text messaging intervention to be evaluated in this study will maximize effects on HIV risk behaviors by incorporating HIV educational content.

### Advances in Technology-Based Interventions for Substance Use and HIV

Mobile phones have the potential to provide an important new delivery medium for behavioral support programs to individuals with drug use disorders. By the end of 2014, the rate of mobile-cellular telephone subscriptions was 98.4 per 100 inhabitants in the United States [27]. There has been a rapid expansion in the use of text messaging, with about 1.92 trillion text messages sent globally in 2014 [28]. The literature describing the use of text messaging as a clinical intervention is rapidly expanding. Text messaging has been used with numerous clinical populations including those with diabetes, obesity, and HIV [29-34], yet the use of these approaches in the treatment of drug users has so far been limited [35,36].

Several studies of text messaging and multimedia intervention programs incorporating text messaging with cigarette smokers have shown improvements in quit rates and attempts and decreases in tobacco use [37-39]. A large randomized trial examined the efficacy of a fully automated digital multimedia smoking cessation intervention which included an intensive text messaging component compared with a control group receiving a self-help booklet; this study found higher point abstinence rates and improved adherence to nicotine replacement therapy relative to the control group [40], with long-term benefits extending through 1-year postintervention.

Mobile phones have been used to disseminate several promising HIV prevention and intervention programs including a text messaging–based sexually transmitted infection and HIV prevention program for adolescents (SEXINFO) [41] and a pilot program employing mobile phone call reminders to improve medication adherence for HIV-infected individuals [34]. Studies to date are typically pilot interventions with small sample sizes, and there is presently little known about the effectiveness of text messaging for HIV-infected populations. Nevertheless, among PWID, a computer-based educational intervention concerning HIV/AIDS was found to have comparable effects on HIV risk behaviors relative to that of a counselor-delivered intervention, and it produced greater retention of HIV-related information [42]. More recently, a text messaging intervention delivering health education and social support in real time to high-risk men who have sex with men effectively reduced methamphetamine use and risky sexual behaviors [36]. Taken together, these data provide reason for optimism that utilizing mobile phone technology could be a promising next step to broadening the availability of cost-effective behavioral interventions for high-need populations such as HIV-infected drug users.

### Aims

This paper describes the methodology leading to the development and pilot testing of a CBT-based text messaging intervention (TXT-CBT) targeting ART adherence and drug use. Although there are features of CBT such as direct therapist feedback concerning therapy homework, for example, that cannot be replicated using a predominantly automated text messaging platform, the TXT-CBT program transports the essential features of CBT for relapse prevention to the technology-assisted platform, with recognition of these inherent limitations. Some of the limitations imposed by a technology-based approach are mitigated by the involvement of a clinician in the TXT-CBT intervention program.

The purpose of this study was to develop, with user feedback, a 12-week CBT-based text messaging treatment program targeting ART adherence and drug use and test its efficacy through a pilot randomized clinical trial. The specific aims of the study were to (1) test the impact of TXT-CBT over and above usual care for HIV on substance use and health care outcomes, (2) evaluate the differential effect of TXT-CBT versus usual HIV care on HIV risk behavior and ART adherence, and (3) examine potential mechanisms of action of TXT-CBT.

The following hypotheses addressed the goals of the study:

- Among substance-dependent adults receiving HIV care, TXT-CBT would yield superior clinical outcomes relative to usual care in reducing substance use and health service utilization during and after treatment. In particular, while some health care utilization may increase among those who benefit from TXT-CBT (eg, use of routine HIV care services), we anticipate reductions in hospitalizations and emergency care services secondary to substance use.
- TXT-CBT would have superior effects on ART adherence and HIV risk behaviors relative to usual HIV care.
- TXT-CBT would have a direct effect on psychological variables that are recognized mechanisms of change in CBT treatment for addicted populations (negative affect, self-efficacy, social support). Specifically, we predicted that reductions in negative affect and cravings and increases in self-efficacy and social support would be positively associated with retention in TXT-CBT and inversely related to substance use during and after treatment.

## Methods

### Study Design

The study began with formative research to develop the intervention. This was followed by a 2-group randomized controlled trial (RCT) in which individuals receiving usual care for HIV were randomly assigned to receive either TXT-CBT or an informational pamphlet concerning HIV and substance use. The trial was conducted and reported in accordance with the Consolidated Standards of Reporting Trials (CONSORT) guidelines [43]. Face-to-face assessments were conducted at baseline and weeks 4, 8, 12, and 24. Phone-based assessments of ART adherence were also conducted at each of these timepoints.

All study procedures were approved by the University of California, Los Angeles Institutional Review Board. This trial is registered at ClinicalTrials.gov [NCT01884233].

### Target/Study Population

Inclusion criteria were (1) age 18 years or older, (2) Diagnostic and Statistical Manual of Mental Disorders, 4th. Edition (DSM-IV) diagnosis of opioid or stimulant dependence, (3) HIV-infected and currently taking ART prescribed within the past 30 days, and (4) currently own a cell phone that can send and receive text messages. Exclusion criteria were (1) lack of proficiency in English; (2) currently homeless (unless residing in a recovery home for which contact information can be provided); (3) dependence on an illicit substance for which medical detoxification is imminently needed; or (4) presence of clinically significant psychiatric symptoms as assessed by the M.I.N.I. International Neuropsychiatric Interview such as psychosis, acute mania, or suicide risk that would require immediate treatment or make study compliance difficult.

### Procedures

Participants were recruited through advertising, word of mouth, study announcement fliers posted in treatment programs and community locations, infectious disease clinics in the community, referrals from local substance abuse treatment and outreach programs, outpatient and inpatient alcohol and drug abuse clinics, primary care providers, local mental health centers, crisis clinics, public service announcements, hospital emergency rooms, and self-referrals. All recruitment materials referred interested persons to clinic phones, which were answered by trained research staff who provided the caller with information about the study and scheduled interested persons for an interview. Interested individuals received a study description and a prescreening prior to an initial interview to begin the informed consent process. Those who wished to participate provided informed consent and began the baseline assessment procedures to confirm eligibility to participate.

### Randomization

Following the prescreening, baseline assessment phase, and confirmation of study eligibility, participants were randomly assigned to group 1, a treatment-as-usual (TAU) condition in which participants received usual care (ie, medical management) for HIV plus a Substance Abuse and Mental Health Services Administration informational pamphlet concerning HIV and substance use (see Multimedia Appendix 1 for the pamphlet content), or group 2, a TAU plus a CBT text messaging condition (TAU+TXT-CBT). Those who were assigned to the TXT-CBT received $20 gift cards monthly over the 3-month intervention phase to offset the costs of an unlimited text messaging plan. Randomization was conducted by a research assistant using a Web-based, automated program implemented by the University of California, Los Angeles, Integrated Substance Abuse Programs Data Management Center. Neither the research staff nor participants were blinded.

### Sample Size

Determination of sample size (proposed n=25 per group) was guided by feasibility and appropriateness to achieve pilot study objectives rather than by statistical power estimates [44,45]. Pilot studies play an important role in providing information for the planning and justification of RCTs in terms of feasibility of intervention protocols and recruitment strategies, feasibility and comprehensiveness of data management and analysis procedures, and exploration of potential effect sizes [46-48]. We anticipated that the sample size (N=50) would provide representativeness of a diversity of HIV-infected substance user characteristics for examining intervention feasibility and produce data characteristics/distributions appropriate to examining data- and analysis-related procedures, variability, and potential effect sizes.

### Intervention Development

The TXT-CBT intervention development process included CBT expert groups, focus groups, and community partners meetings conducted to obtain input for the content of the intervention and a logic model for the TXT-CBT intervention developed based upon rules that supported tailoring the intervention content over the 12-week period. Concurrently, text messages for the TXT-CBT library were written and categorized by outcome target (substance use, HIV risk behaviors, ART adherence). Written materials provided to the participants were also developed. A participant TXT-CBT manual was developed including instructions on how to use the text messaging system and detailing the specific psychological skills to be learned and rehearsed upon participation in the intervention. The beta version of the TXT-CBT text messaging system was developed and a pilot study of the beta version was conducted among HIV-positive, opioid-dependent adults (n=10) receiving HIV care. For this pilot test, all planned measures for the randomized trial were collected to determine feasibility of the protocol. Participants received qualitative data collection calls weekly during their participation in TXT-CBT to gather information concerning the functionality of the program, address any technical difficulties they might experience, and determine whether any of the messaging content was unacceptable or in need of revision. Based upon the pilot study findings, the intervention was refined and accompanying materials finalized in preparation for the pilot RCT.

### Intervention Content

The intervention comprised one CBT-based face-to-face counseling session with a master’s level clinician followed by 12 weeks of daily text messages. The messages included medication reminders plus 2 or 3 additional messages on the topic of addiction recovery and associated risk behaviors. Participants selected the desired frequency and time at which to receive their medication reminder messages. Participants enrolled thus far typically require 1 or 2 medication reminders daily. As such, participants receive 4 or 5 text messages daily. Of the 14 to 21 messages per week that are not medication reminders, approximately half of the message content pertains to CBT skills for drug relapse prevention with the remaining half split evenly between content concerning common HIV risk behaviors and ways to reduce them and the importance of ART adherence coupled with behavioral strategies to promote adherence.

During the clinician-delivered CBT session, a number of variables upon which the intervention was tailored or individualized were identified, including the top 3 barriers to ART adherence for the individual, with corresponding coping skills training; the participant’s first name; specific times of day at which medication reminders are needed; and motives for quitting substance use.

Content had both informational and interactive components to model the balance between psychoeducation and counseling in traditional clinician-delivered CBT. The remaining texts provided psychoeducation, tips, suggestions, and reminders to facilitate the use of cognitive and behavioral strategies to prevent substance relapse, improve ART adherence, and reduce HIV risk behaviors. Relapse prevention messages comprised 50% of those sent to participants each week (see Textbox 1). The 12-week intervention is staged such that each week a new specific coping skill was introduced:

1. Behavioral techniques such as scheduling and engaging in alternative rewarding activities to prevent boredom and maintain a sober lifestyle

2. Opioid withdrawal symptoms, similarities between stress and withdrawal symptoms, and coping techniques to prevent relapse in the face of these symptoms

3. Stress as a relapse precipitant, with descriptions of stress management techniques in the form of easy-to-implement tips

4. Common triggers to opioid relapse with suggestions on how to eliminate or minimize triggers

5. Relapse analysis practice, in which an example of a prior relapse is described to identify its precipitants

6. Goal-setting with interactive messages used to help participants specify goals that are incompatible with substance use

7. Family and social support for abstinence; emphasizing avoiding isolation

8. Impact of addiction and the recovery process on important relationships, with suggestions and tips regarding family roles in treatment

9. Behavioral lifestyle changes that support recovery

10. Decisions about other drug use (eg, marijuana)

11. Motivation to remain abstinent

12. Individualized relapse prevention planning, with interactive messages to identify components of a personalized relapse prevention plan (eg, contact information for support, preferred behavioral strategies such as exercise, and cognitive techniques such as reviewing reasons for quitting).

**Behavioral targets and examples of text messages.**

ART adherence

- Taking ur medicine exactly as prescribed will help u 2 get the most benefit from it.
- By taking ur meds on schedule, ur keeping ur viral load low and ur CD4 count right where it needs 2 be! It's worth the effort!!
- If u slip up, the best choice is 2 get right back 2 taking ur meds ASAP!

Relapse prevention

- Which feelings R triggers 4 U? Txt them to us. Text EXAMPLE for examples of common ones.
- Keep ur life in balance! Have fun with family or friends, be kind to yourself, and always fill in free time with activities!
- Relapse is a slow process that begins long b4 U actually pick up or start drinking. It helps to watch 4 early signs. Do u know ur signs?

HIV risk behaviors

- Even if u r taking meds and lower ur viral load, there’s still a risk that u will transmit HIV to a partner during unprotected sex. Always use a condom!
- If U get infected with a different strain of HIV, this is called “superinfection.” It can make u sicker bc it may b harder 4 ur immune system 2 control.
- Safe sex takes two! Make sure 2 talk to ur partner about using condoms beforehand.

#### HIV Risk Reduction Content

Text messages targeting HIV risk reduction were developed based on key constructs in the Cognitive-Social Health Information Processing model [49] coupled with a recently developed text messaging–based intervention targeting HIV risk behaviors among methamphetamine users [35]. Psychoeducational messages regarding HIV transmission, medical consequences, treatments, risky drug use behaviors, sexual risk behaviors, and ways to prevent transmission were included. HIV risk reduction messages comprised 25% of those sent to users each week.

#### Adherence Content

Messages concerning HIV medication adherence were based on the CBT program Life-Steps [13], which comprised education, problem solving, and rehearsal strategies to help patients develop better skills for adhering to HIV treatment. Content following the Life-Steps model was tailored to each individual based on the top 3 barriers to adherence identified in a meeting with a CBT clinician at baseline. Content may have included education about adherence, scheduling, cue control strategies including the use of the mobile phone alarm, adaptive thoughts about adherence, and improving communication with medical providers [11]. Adherence messages comprised 25% of those sent to each user weekly.

#### Text CRAVE

In addition to the programmed delivery of daily messages, participants had the option to send a CRAVE text that generated programmed feedback in the form of advice or suggestions when they experienced a craving [50]. In this regard, the TXT-CBT intervention provided a near real-time medium to facilitate the use of social support and feedback, key elements of CBT model, for coping with the craving without using. To keep the messages novel and nonrepetitive, participants rarely (if ever) received the exact same message more than once throughout the duration of the study.

#### Treatment As Usual

All participants were receiving usual care for HIV, which comprised pharmacological management by a physician in an infectious disease clinic setting. Generally, participants were scheduled for monthly visits with their physician and quarterly assessment of viral load and CD4 counts.

### Clinician Involvement

Apart from the single face-to-face CBT session, TXT-CBT participants may have had contact with the CBT clinician in one of two contexts. In the text CALL function, participants were prompted to text the word CALL if they needed more instruction about how to implement a particular coping skill that they had planned to use to facilitate ART adherence. When a text CALL was received, a clinician called the participant back and provided counseling to assist with the specific skill for which guidance was needed. In the text LIFELINE function, participants could text the word LIFELINE if they needed to speak to a clinician during business hours. All participants were able to use this function a total of three times during the 12-week intervention. For both text CALL and text LIFELINE functions, prior to receiving a call back from a clinician, participants received an automated response to remind them of the hours during which the clinician is available and were instructed to call 911 or go to an emergency room for any urgent matters beyond these hours or that required an immediate response. If the text was received during business hours, the clinician called the participant on the same day. For any participant who used the LIFELINE function three times or more, the protocol specified that a licensed clinician on the research team was to be consulted to determine whether a treatment referral was indicated and/or whether the participant could continue to participate in the study.

### Technical Support

Participants received weekly calls from the research staff to collect qualitative data concerning the perceived helpfulness of the TXT-CBT intervention and address any technical difficulties they may have been experiencing. A research assistant reviewed the text messaging logs regularly to monitor whether text messages were being sent on time and whether participants were engaging in texting. If participants wished to disenroll from the study they could text the keyword STOP.

### Outcome Measures

See Table 1 for an overview of the outcome measures. Primary outcomes included ART adherence, substance use, and HIV risk behaviors. ART adherence was assessed by phone using an unannounced pill count (UPC) procedure. All other outcome measures were administered in face-to-face interviews, with some clinician-administered measures and the majority of the assessments conducted using a Web-based data entry system. Participants were compensated in gift cards as follows: $40 for the baseline assessment, $20 for each monthly face-to-face assessment, and $20 for each phone-based pill count procedure. Additionally, for bringing in photocopies of viral load and CD4 count assay reports from their primary HIV care provider, participants were compensated $20 at baseline (BL), treatment end, and follow-up.

### Primary Outcomes

#### Substance Use

Substance use was assessed using urine drug screens, the Addiction Severity Index, and the Timeline Followback (TLFB) method. Urine drug screens were collected at baseline and weeks 4, 8, 12, and 24 using temperature-controlled test cups. A US Food and Drug Administration–approved one-step, rapid dip drug test is used (CLIAwaived Inc). The urine drug screens tested for the presence of amphetamines, benzodiazepines, methadone, cocaine, methamphetamine, barbiturates, oxycotin, opiates, and marijuana.

The Addiction Severity Index [51], collected at baseline, treatment end, and follow-up, was used to assess psychiatric and substance use disorder severity and other domains of functioning. Composite scores (alcohol and drug, psychiatric, legal, family, and employment) will be contrasted between groups at baseline to evaluate the effectiveness of randomization. Drug use severity composite scores will be used as a covariate in the analysis of differential treatment effects on outcomes.

The TLFB, a calendar-assisted structured interview [52] with demonstrated validity in substance treatment samples [53], was used to assess drug use in the preceding 90-day period at baseline and follow-up and preceding 30-day drug use at weeks 4, 8, and 12. The TLFB was used to calculate self-report substance outcome variables including percent days abstinent as reported in Project MATCH [54]. Time to first relapse was also evaluated using the TLFB. As in prior work [55], relapse was defined as the first drug use following 7 consecutive days of abstinence after the initiation of treatment.

#### HIV Risk Behavior

The Behavioral Risk Assessment [56] measured HIV risk behavior. This instrument recorded data on participants' sociodemographic characteristics (gender identity, sexual identity, age, race/ethnicity, HIV status, educational attainment, housing status), substance use in the previous 30 days (injection and noninjection drug use and safe needle use protocol), number and gender of sexual partners in the previous 30 days (main, casual, anonymous, exchange and male, female, male-to-female preoperative transgender, female-to-male preoperative transgender), and details about the participants' three most recent sexual encounters within the previous 12 months (partner type, number of partners in the encounter, HIV status of partner[s], sexual activities during the encounter, substance use by participant and partner[s], location of sexual encounter).

#### ART Adherence

ART adherence was assessed using both phone-based UPCs and viral load data. Both home- and phone-based UPCs have been shown to be reliable and valid measures of HIV treatment adherence, yielding comparable data to electronic medication monitoring [57,58] and significant correspondence with plasma viral load [59]. At the initial research visit, participants were trained by an intake assessor and a phone-based pill counter on the telephone in procedures for conducting UPCs. Training focuses on instructing participants to organize and count their pills. The first UPC was conducted within 1 week of the training, with subsequent monthly UPCs at unannounced times during and after the intervention through follow-up at week 24. All UPCs were conducted for each ART medication the participant is taking. Pharmacy information from pill bottles was also collected to verify the number of pills dispensed between calls. A medication adherence score was then calculated as the ratio of pills counted to pills prescribed, taking into account the number of pills dispensed. Using this method, the adherence score represents the percentage of pills taken as prescribed averaged across medications [60].

Viral load served as a biological indicator of adherence. Consistent with the typical frequency with which viral load is assessed in clinical settings, data concerning viral load was collected at baseline, treatment end, and follow-up. Participants either provided us with a copy of their latest viral load results or signed a medical release of information allowing us to obtain their viral load results directly from their medical provider.

**Table 1:** Key variables and measurements.

	Variables	Measurements	Screen	BL	Week 4	Week 8	Week 12	Follow-up
**Primary outcomes**								
	Substance use	Addiction Severity Index^b^		x			x	x
		Urine drug screen^b^		x	x	x	x	x
		Timeline Followback^b^		x	x	x	x	x
	HIV risk behavior	Reback Behavioral Risk Assessment^c^		x			x	x
	ART adherence	Unannounced pill counts^b^		x	x	x	x	x
		Viral load/CD4^b^		x			x	x
**Secondary outcomes**								
	Medication compliance	Self-report^c^		x	x	x	x	x
	Behavioral strategies	Self-report^c^		x				x
	Depression	PHQ-9^c,d^		x	x	x	x	x
	Anxiety	OASIS^c,e^		x	x	x	x	x
	Drug-taking self-efficacy	Drug-Taking Confidence Questionnaire^c^		x			x	x
	Health-related quality of life	SF-12^c,f^		x			x	x
	Readiness for change	SOCRATES-D^c,g^		x				
**Covariates**								
	Demographics	Self-report^c^	x					
	Psychiatric diagnosis	M.I.N.I. International Neuropsychiatric Interview^b^	x					
	Concomitant medications	Self-report^b^		x	x	x	x	x
	Ancillary treatments	Self-report^c^		x	x	x	x	x
**Participant satisfaction** ^a^								
	Participant satisfaction	Weekly qualitative calls^b^			x	x	x	
	Participant adherence	Message exposure^b^			x	x	x	

^a^Only for participants randomized to the TXT-CBT group.

^b^Research assistant–administered.

^c^Participants used a Web-based data entry system.

^d^Patient Health Questionnaire.

^e^Overall Anxiety Severity and Impairment Scale.

^f^Short Form Health Survey.

^g^Stages of Change Readiness and Treatment Eagerness Scale.

### Secondary Outcomes

#### Self-Reported Medication Adherence

Self-reported adherence was assessed monthly using a brief 3-item adherence survey developed by Kalichman et al [61]. Using a visual analogue rating scale, participants were asked to indicate their best guess, ranging between 0% and 100%, about how much of their HIV medication they have taken in the past week and in the past month. They were also asked how confident they were that they can take all of their HIV medications in the next week.

#### Behavioral Strategies for Adherence

From a 25-item behavioral strategies checklist developed by Kalichman et al [60], participants were asked to indicate how often they have used these strategies during the past 3 months. Answer choices are never, sometimes, often, very often, and always. Examples of strategies included in the questionnaire are “used a pill box organized by day of the week” and “used bedtime as a reminder to take medications.”

#### Depression

The Patient Health Questionnaire (PHQ-9) [62,63] is a self-administered, 9-item questionnaire used to assess depression. It includes DSM-IV depression criteria as well as other major depressive symptoms. The PHQ-9 is a reliable and valid measure of depression [62,63]. Participants were asked to report “how often they have been bothered by any of the following problems” with response choices ranging from “not at all” to “nearly every day.”

#### Anxiety

The Overall Anxiety Severity and Impairment Scale (OASIS) was used to assess severity and impairment associated with anxiety [64]. It is a 5-item measure that is valid across anxiety disorders, for multiple anxiety disorders, and with subthreshold symptoms [64]. Participants were asked to identify which answer best describes what they have been feeling in the past week. Three questions have answer choices that range from “none” to “extreme,” and two range from “none” to “constant” anxiety.

#### Drug Taking Self-Efficacy

Drug taking self-efficacy was measured using the Drug-Taking Confidence Questionnaire [65], which measures confidence in avoiding alcohol and other drugs (abstinence goal) or heavy use (moderation goal) across the 8 high-risk categories in Marlatt’s taxonomy of relapse precipitants [66]. Participants imagined themselves in 8 specific situations and rated confidence in resisting the urge to drink heavily or use a specific drug.

#### Health-Related Quality of Life

The health status questionnaire [67,68] measures mental and physical functioning as well as overall health-related quality of life. All 12 questions come from the 36-item Short Form Health Survey (SF-36) [67,68]. Participants were asked whether their daily activities are impacted as a result of their physical health or emotional problems.

#### Readiness for Change

The Stages of Change Readiness and Treatment Eagerness Scale (SOCRATES-D) is a 19-item scale used to assess readiness and motivation for change in drug users [69]. The SOCRATES instrument has 3 subscales: Recognition, Ambivalence and Taking Steps. Participants are asked to rate how much they agree or disagree with a statement “right now.” Possible answers range from “strongly disagree” to “strongly agree.” Examples of statements are “I really want to make changes in my use of drugs,” “sometimes I wonder if I am in control of my drug use,” and “I am actively doing things now to cut down or stop my use of drugs.”

### Covariates

Demographic variables, psychiatric diagnosis, ancillary treatments, and message exposure (participant adherence to TXT-CBT) were examined as potential moderating or mediating variables.

### Participant Satisfaction With TXT-CBT

Participant satisfaction with the TXT-CBT intervention was assessed using qualitative weekly phone-based interviews in which those assigned to the experimental group were asked a series of scripted questions concerning the perceived helpfulness of the intervention in that week. Participants were queried concerning any messages that stood out as particularly helpful or that they might have disliked and whether and how the intervention helped them to change behaviors in the key domains of interest (ie, ART adherence, drug use, and/or other risk behaviors).

### Statistical Analyses

Analyses in the pilot study will allow basic description of the sample in terms of distributional characteristics, variability, and missing data as well as examination of possible effect sizes in comparing the groups. Preliminary analyses will include descriptive statistics and confidence intervals, computation of scale scores, and assessment of reliability. Exploratory analyses will use a random effects regression approach to examine group differences across the repeated observations (baseline, treatment end, and follow-up) as specified in each of the study aims. This type of model accommodates the (potentially correlated) repeated measures and allows for inclusion of covariates (ie, subject characteristics on which the groups differ at admission) and additional repeated observations for some measures (eg, viral load at baseline, treatment end, and follow-up). The pilot sample size of 25 per group should allow detection of an effect size of approximately d=.7 in differential change in outcomes from baseline to follow-up, assuming a moderate correlation of 0.50 over time and allowing attrition of up to 20% (power=.80, one-tailed alpha=.05). This detectable effect size would translate to a differential change of about 19 points on the ART adherence composite score assuming an average (across time) pooled (across groups) standard deviation of about 27, as found by Safren [11] for a specialized CBT intervention for HIV-infected individuals. Safren et al found a differential change of approximately 25 points.

Separate analyses will address each measure in aims 1, 2, and 3. For example, analyses for aim 2 will include as dependent variables urine toxicology results for opioids and stimulants, ART adherence scores, health-related quality of life scores from the SF-12, and select items from the ASI indicating number of days in the past month utilizing health services. Analyses for aim 3 will include correlations between changes in self-efficacy (Drug-Taking Confidence Questionniare) and treatment retention and substance use during and after treatment.

## Results

Currently, data collection is ongoing and is expected to be completed in July 2016. We expect that analysis and results will be available by September 2016.

## Discussion

### Principal Findings

In this clinical trial protocol, we present the study design of a two-phase intervention development and evaluation project in which we (1) developed, with user input, a 12-week CBT-based text messaging intervention targeting ART adherence and drug use in an adult addicted population with HIV and (2) pilot tested the intervention in an RTC. We hypothesize that TXT-CBT will directly impact ART adherence and substance use, over and above the effect of usual care for HIV, relative to those who receive usual HIV care in conjunction with an informational pamphlet concerning HIV, ART adherence, and substance use. Exploratory analyses will be conducted to examine process changes in TXT-CBT and their associations with outcomes. We expect that mechanisms of action demonstrated in prior work examining the key ingredients of face-to-face CBT (eg, self-efficacy) will also account, at least in part, for any therapeutic effects of TXT-CBT on the target clinical outcomes in this study.

### Limitations

The major limitation of the study design is the absence of a control condition matched for time and attention. Likewise, data collection procedures that are specific to the active TXT-CBT condition, such as qualitative data collection efforts to ascertain perceived acceptability and helpfulness of the intervention, were not balanced between conditions and could potentiate the effects of the TXT-CBT intervention. Nevertheless, this initial investigation is designed to establish the acceptability and preliminary outcomes of the TXT-CBT intervention; if the current hypotheses are supported, a text messaging control condition will be developed as part of a larger, fully powered RCT. Likewise, the sample size limits the statistical power for detecting effects of the intervention; nevertheless, as a pilot trial, the proposed N is appropriate for meeting the study objectives.

### Strengths

This is the first trial to our knowledge that uses a theory-based intervention approach to the treatment of addiction via a text messaging platform. As such, rather than implementing a single behavioral strategy (eg, reminders or motivational sayings), the TXT-CBT provides a therapy program that draws its content from evidence-based addiction and medication adherence interventions. Second, we focus on a well-defined population (ie, HIV-infected adults with substance use disorders) with an urgent need for accessible, low-cost intervention approaches to reduce risk factors for the spread of HIV infection (including drug use and poor ART adherence). Third, we involved the users closely in the intervention development process to optimize the likelihood of acceptability and participant retention in TXT-CBT. Finally, by investigating mechanisms of action of TXT-CBT, this study is expected to advance our understanding of the psychological processes by which text messaging interventions effect behavior change.

### Conclusions

We present the methodology germane to the development and pilot testing of TXT-CBT, a theory-based intervention using a technology-based text messaging platform for HIV-infected adults with substance use disorders. By providing support to maximize HIV treatment regimen adherence and reinforce coping skills to prevent relapse to substance use, TXT-CBT may provide a promising, cost-effective, and easily deployable strategy for the treatment of substance users who are HIV-infected.

## Appendix

Multimedia Appendix 1 Substance Abuse and Mental Health Services Administration Pamphlet. Drugs, Alcohol and HIV/AIDS: A Consumer Guide.

## Abbreviations

ART: antiretroviral treatment
CBT: cognitive behavioral therapy
DSM-IV: Diagnostic and Statistical Manual of Mental Disorders, 4th Edition
OASIS: Overall Anxiety Severity and Impairment Scale
OST: opioid substitution therapy
PHQ-9: Patient Health Questionnaire–9
PWID: people who inject drugs
RCT: randomized controlled trial
SEXINFO: Sexually Transmitted Infection and HIV Prevention Program for Adolescents
SF-36: 36-item Short Form Health Survey
SOCRATES-D: Stages of Change Readiness and Treatment Eagerness Scale
TAU: treatment-as-usual
TLFB: Timeline Followback
TXT-CBT: cognitive behavioral therapy–based text messaging intervention
UPC: unannounced pill counts
