# Supplementary material for: A Cognitive Behavioral Therapy–Based Text Messaging Intervention Versus Medical Management for HIV-Infected Substance Users: Study Protocol for a Pilot Randomized Trial
Source: JMIR Res Protoc. 2016 Jun 24;5(2):e131. doi: 10.2196/resprot.5407 (PMC4938885; doi:10.2196/resprot.5407)
Supplement: Multimedia Appendix 1 [file resprot_v5i2e131_app1.pdf]

Drugs, Alcohol and HIV/AIDS

A Consumer Guide

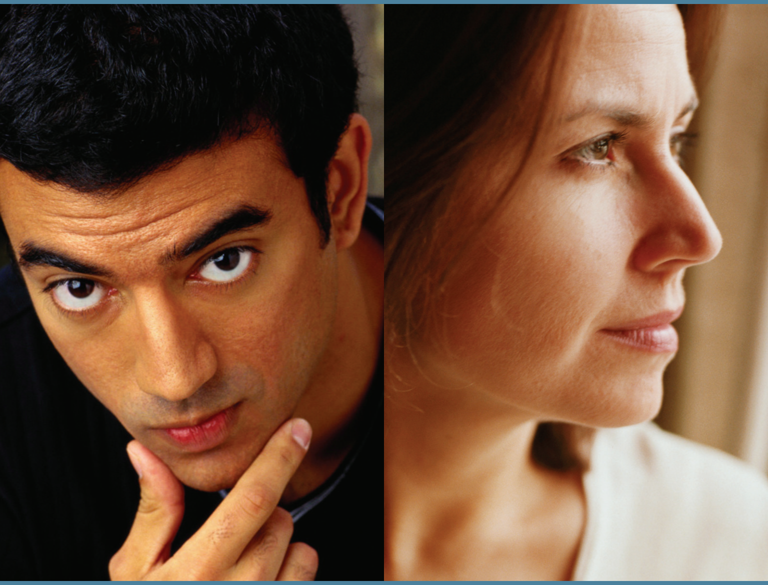

DEPARTMENT OF HEALTH & HUMAN SERVICES · USA

Substance Abuse and Mental Health Services Administration
SAMHSA
www.samhsa.gov • 1-877-SAMHSA-7 (1-877-726-4727)

Drugs & Alcohol

What do drugs and alcohol have to do with HIV?

Drug and alcohol use can lead to risky behavior that can increase your exposure to HIV/AIDS. For example, using or sharing needles or other drug materials, like rinse water or cotton, increases your chances of becoming infected with HIV.

What if I already have HIV?

There are different types of HIV called strains. Even if you already have one strain, using drugs puts you at risk for being infected with a different one. If you are pregnant, you could even infect your unborn baby.

What if I can't stop using drugs or alcohol?

Don't be afraid or ashamed to ask for help. Call the Substance Abuse and Mental Health Services Administration's [SAMHSA] National Helpline (1-800-662-HELP [4357]) for help in getting treatment. It can be your first step toward recovery.

If you decide to get drug or alcohol treatment in addition to HIV treatment, let your current doctor know. He or she can work with your drug or alcohol treatment provider to make sure the two types of treatment work together.

Did you know?

- **Drug abuse behavior plays the single largest role in the spread of HIV infection in the United States today.**

Source: National Institute on Drug Abuse, March 2005.

HIV/AIDS

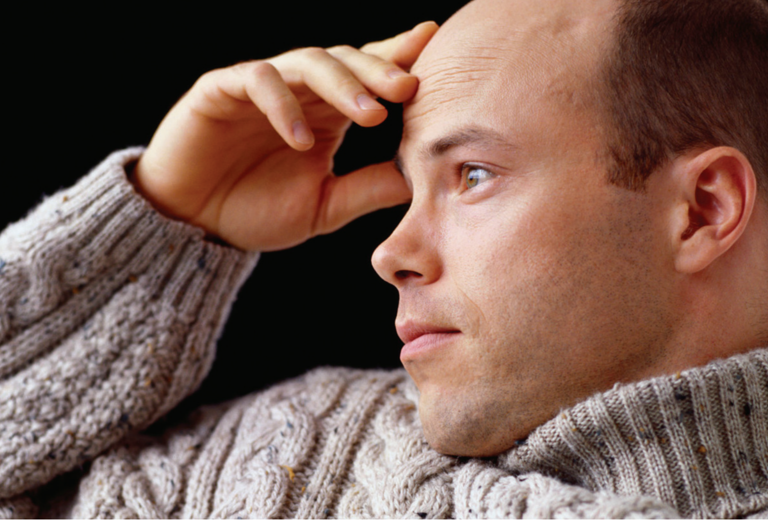

*Half of all new HIV infections now occur among injection drug users.**

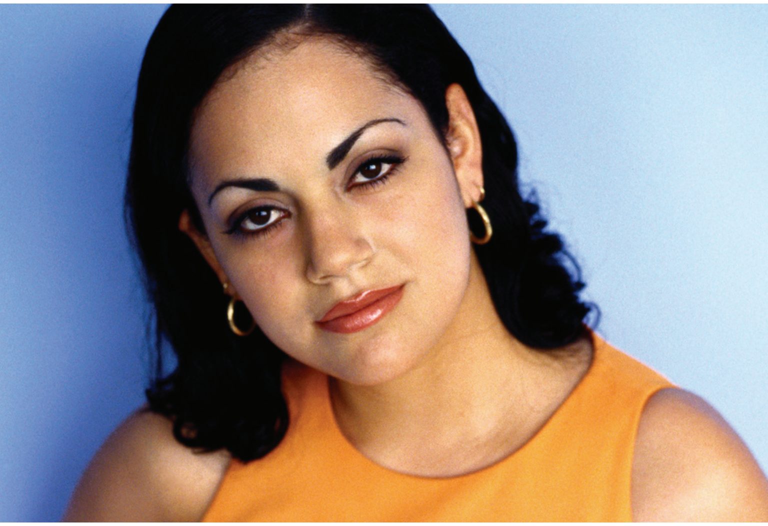

What if I can't handle all of this alone?

Don't worry you don't have to. Listed on the back of this brochure are hotlines and Web sites that can help you find good health care, the treatment you need, and even a place to live if you need one. Call the toll-free number or visit the Web site of your choice today. It could positively change your whole life.

* National Institute on Drug Abuse, 2003

# You are not alone. Help is available.

Call These Toll-Free 24-Hour Phone Numbers

SAMHSA's National Helpline
Línea Directa (agencia) para Tratamiento de las Drogas y el Alcohol
1-800-662-HELP (4357)

National AIDS Hotline
1-800-342-AIDS (2437)

Línea Directa (agencia) Nacional del SIDA
1-800-344-SIDA (7432)

Gay Men's Health Crisis Hotline
1-800-AIDS-NYC (243-7692)

Or Visit These Web Sites

Substance Abuse Treatment Facility Locator
http://www.findtreatment.samhsa.gov

Centers for Disease Control and Prevention
http://www.cdc.gov/hiv/default.html

AIDS*info*
http://www.aidsinfo.nih.gov

Office of National AIDS Policy
http://www.whitehouse.gov/administration/eop/onap/

American Foundation for AIDS Research
http://www.amfar.org

Or check the box below to see if a treatment center near you has listed its address and/or phone number.

The people who appear in this brochure are models and are used for illustrative purposes only. Do not reproduce or distribute this publication for a fee without specific, written authorization from the Office of Communications, Substance Abuse and Mental Health Services Administration (SAMHSA), U.S. Department of Health and Human Services.

This brochure was created to accompany the publication *Substance Abuse Treatment for Persons With HIV/AIDS,* #37 in SAMHSA's Treatment Improvement Protocol (TIP) Series. The TIP series and its affiliated products may be ordered from SAMHSA's Publications Ordering Web page at http://store.samhsa.gov. Or, please call SAMHSA at 1-877-SAMHSA-7 (1-77-726-4727) (English and Español).

HHS Publication No. (SMA) 15-4127
Printed 2006
Revised 2007, 2008, 2011, 2014, and 2015
